# Why simulations are appropriate for evaluating Qualitative Comparative Analysis

**DOI:** 10.1007/s11135-015-0251-8

**Published:** 2015-07-30

**Authors:** Ingo Rohlfing

**Affiliations:** Bremen International Graduate School of Social Sciences (BIGSSS), University of Bremen and Jacobs University Bremen, Bremen, Germany

**Keywords:** Qualitative Comparative Analysis, Monte Carlo simulation, sensitivity analysis, case knowledge

## Abstract

QCA has recently been subject to massive criticism and although the substance of that criticism is not completely new, it differs from earlier critiques by invoking simulations for the evaluation of QCA. In addition to debates about the meaning of the simulation results, there is a more fundamental discussion about whether simulations promise any relevant insights *in principle*. Some voices in the QCA community reject simulations per se because they necessarily lack case knowledge. As a consequence, the debate is at an impasse on a metalevel because critics of QCA rely on simulations, the results of which some QCA proponents refuse to consider as insightful. This article addresses this impasse and presents six reasons why simulations must be considered appropriate for evaluating QCA. I show that if taken to its conclusion, the central argument against simulations undermines the need for running a truth table analysis in the first place. The way forward in this debate should not be about whether simulations are useful, but how to configure meaningful simulations evaluating QCA.

## Introduction

Since its introduction in 1987, Qualitative Comparative Analysis (QCA) has received as much appraisal as it has been the target of criticism. The most recent, broad wave of criticism does not differ in its substance from previous discussions, but it invokes *simulations* for the evaluation of QCA (e.g., Seawright 2014). While simulations have been used before (Marx and Dusa 2011), Skaaning 2011), the breadth of their application is unprecedented in the history of QCA.

For the purpose of my article and in line with the general usage of this term, I broadly define ‘simulation’ as the manipulation of the data generation process or the data analysis stage with the goal of determining how a certain element of the analysis is affected. In quantitative research, for example, we might be interested in the size of the standard error as one element of regression output. In QCA, we can be interested in the robustness of QCA solutions (Krogslund et al. 2015) or consistency scores (Braumoeller forthcoming). Simulations can be at least partially based on empirical data or exclusively on hypothetical data and involve Monte Carlo Simulations or a single simulation (Krogslund et al. 2015; Lucas and Szatrowski 2014). I refer to the latter as one-shot simulations in the following.

Regardless of what type of simulation is implemented, some pro-QCA voices in the debate refute the realization of any simulation for the evaluation of QCAs performance. This rejection of simulations is founded on the claim that QCA is a case-based method and that simulations lack case knowledge (Olsen 2014; Ragin 2014). In fact, there is disagreement among pro-QCA scholars whether simulations are good and some endorse them, at least implicitly (Vaisey 2014). Up to now, however, there is no explicit discussion about reasons making simulations useful for the assessment of QCA.

I argue that, for multiple reasons, the rejection of simulations for the evaluation of QCA is untenable. Some of these reasons refer to method-driven analyses of QCA, while others pertain to features and challenges of specific empirical QCA studies. The first three of my arguments are of general nature and are independent of the algorithm that is used for the truth table analysis and any design feature in QCA.^1^ The first point is the most fundamental and aims at the heart of the claim that case knowledge renders insights from simulations superfluous (Sect. 2). I demonstrate that if one had perfect knowledge about all cases (regardless of whether this is feasible in practice), the truth table analysis invoking an algorithm, which is constitutive for QCA (Ragin 1987, Chap. 6), would become superfluous. A lack of case knowledge in simulations is not a valid charge against them because this lack is a prerequisite for doing a truth table analysis in the first place.

*Modeling uncertainty* is pervasive in empirical research and pertains in QCA, for example, to the choice of the proper calibration thresholds and consistency threshold (Skaaning 2011). Modeling uncertainty motivates the second reason for running simulations in QCA. Second, in any empirical study, it is mandatory to perform a robustness analysis so as to discern whether the QCA solution is sensitive to a modeling decision, which I show to be the same as running a simulation (Sect. 3).

The following three points are in the broadest sense data-related and not tied to modeling decisions and uncertainty. The common element of the three data-related points is that the observed data and cases we *use* for causal inference is only a subset of the cases we *make* inferences about. On this basis, the third issue is linked to the presence of *limited diversity* in QCA (Sect. 4.1). When limited diversity is given and we rely on the Quine-McCluskey algorithm (Ragin 1987, Chap. 6), we have to invoke counterfactuals for the generation of the intermediate and parsimonious solution. Since we face inherent uncertainty about what the correct counterfactuals are, simulations are a proper tool for assessing the dependence of a QCA solution on the counterfactuals that we make. Fourth, I demonstrate that *sampling* is the second data-related issue that warrants simulations because a sample is, by definition, only a subset of the theoretically relevant population (Sect. 4.2). Fifth, a similar line of reasoning holds for *missing data* because the non-missing data is only a subset of the full data we would like to analyze (Sect. 4.3).

The final, sixth point takes an exclusively method-driven perspective on QCA that abstracts from a specific empirical analysis (Sect. 5). In such a perspective, Monte Carlo simulations are the only way of determining how QCA performs *in general*. A corollary of the discussion is that evaluations of QCA based on a one-shot simulation cannot produce any relevant information about the performance of QCA (Lucas and Szatrowski 2014).

The current controversy about QCA makes it necessary to emphasize what I do and do not argue for. First, case knowledge is useful in QCA and, more generally, any empirical research, including quantitative and experimental studies (Dunning 2008, Chap. 7). Solid case studies might reduce uncertainty and enhance QCA and a truth table analysis. However, it is unlikely case studies can every reduce all uncertainty and, if they could, would dispense with the truth table analysis. Second, my support of simulations for the evaluation of QCA does not imply anything about the quality of the simulations that have been done so far and their results. *How* simulations should be done is an important question to consider, but it differs from my goal of showing *that* simulations are valuable. Moreover, it should be understood that simulations do not diminish uncertainty, but serve to assess the consequences of uncertainty on the robustness and validity of QCA results. Third, saying that simulations are useful in principle does not mean that they should always be applied. Depending on the topic under scrutiny, it might be preferable to approach an issue such as case-wise deletion from an analytical perspective (Thiem 2014).

In this spirit, this paper seeks to contribute to the development of a basis for simulations in QCA. If all parties involved in the debate about QCA would agree on the potential value of simulations, we would share common ground from which we could discuss how to design simulations and what they tell us about the ability of QCA to generate valid results.

## Perfect case knowledge dispenses with truth table analyses

The case-orientation of QCA is foundational because QCA was proposed as a middle road between case studies and quantitative analysis (Ragin 1987, Chap. 1). Case knowledge enters QCA at multiple stages and with different functions before the truth table is processed with an algorithm (Ragin 1987, Chaps. 5–7) and afterwards (Schneider and Rohlfing 2013). For the argument I make in this section, it is only important to reflect on case knowledge that overlaps with the reason for which a truth table is analyzed, which is the generation of a QCA solution that can be causally interpreted (Ragin 1987, Chap. 5, 6).^2^ Besides that it is a salient topic in the debate, the reason is that this setup is favorable for the critics of simulations; if there is one issue on which case knowledge could be brought to bear before the truth table analysis and the charge against simulations could be valid, it is the detection of causally irrelevant conditions, i.e., the overspecification or overfitting of truth tables. Other modeling decisions such as the specification of calibration and consistency thresholds are as challenging as the construction of truth tables, but these are decisions that, in my view, can be validated empirically with neither case studies nor with a truth table analysis.

The two non-exclusive options for constructing a truth table are theory and empirical insights derived from prior research (Amenta and Poulsen 1994). Regardless of how the truth table is derived, a problem that can occur in its analysis is *overspecification* (Schneider and Rohlfing 2014).^3^ Overspecification means that too many conditions have been included in the analysis, i.e., some conditions are not causally related to the outcome in fact.^4^ Simulations have been used to determine whether the Quine–McCluskey algorithm can single out superfluous conjuncts in overspecified truth tables and produce the correct solution (Krogslund et al. 2015; Lucas and Szatrowski 2014; Seawright 2014).^5^

Simulations of the consequences of overspecification on the validity of QCA solutions have been rejected with the argument that no case knowledge is involved in the analysis of hypothetical data, thus ignoring a constitutive feature of empirical QCA studies (Olsen 2014; Ragin 2014). This argument entails the claim that we can determine superfluous conditions in case studies prior to the truth table analysis and avoid the problem of overspecification. Indeed, the literature on single-case counterfactuals and comparative case studies has a long history of developing rules for determining the causal relevance of a condition and could be invoked for this purpose in QCA (Rohlfing 2012, Chap. 4, 7).

In principle, single case-studies and pairwise comparisons are beneficial for screening candidate conditions and to avoid the construction of overspecified truth tables. However, if we run case studies and assume that we correctly separate all non-causal conjuncts from causal ones, what role is left for the truth table analysis? The answer is “none”. The rationale for running an algorithm on the truth table is to determine whether a conjunct is redundant. When we use the Quine–McCluskey algorithm (Ragin 1987, Chap. 6), we follow the one-difference rule for separating redundant from non-redundant conjuncts (Baumgartner 2013). A conjunct is redundant when two conjunctions share the outcome and all conjuncts except one. In this setting, the conjunct that varies across the two conjunctions is redundant and the two conjuncts are simplified to one that only displays all invariant conjuncts.

For example, the conjunctions *ABC* and $AB\lnot {C}$ show that the conjunct *C* is redundant and are simplified to *AB* if both are linked to the outcome *Y*. Along these lines, the Quine–McCluskey algorithm serves to discern superfluous conditions, which is exactly the same goal that we attach to case studies done before the truth table analysis. Claiming that simulations on the consequences of overspecification are pointless because case knowledge in an actual QCA study will identify all superfluous conjuncts shifts the goal and achievements of the truth table analysis to the preceding case study stage, which, in turn, renders the truth table analysis irrelevant.

A simple hypothetical example illustrates this point. Assume we are interested in the determinants of current party position change (*CC*) between two elections and that a review of the literature allows us to focus on three conditions (Adams 2012): median voter position change (*MV*), median party supporter change (*MS*) and the party position change between the second-to-last and the previous elections (*PC*). I assume that each of the eight three-way conjunctions is observed once, i.e., there is full empirical diversity (I turn to limited diversity below).^6^ Table 1 contains the truth table with outcome values assigned to each configuration based on the hypothetical membership of the respective case in the outcome.

**Table 1:** Hypothetical truth table

Row	Median voter (*MV*)	Median supporter (*MS*)	Previous change (*PC*)	Current change (*CC*)	n
A	1	1	1	1	1
B	1	1	0	1	1
C	1	0	1	1	1
D	1	0	0	1	1
E	0	1	1	0	1
F	0	1	0	0	1
G	0	0	1	0	1
H	0	0	0	0	1

Now imagine that we realize case studies before the truth table analysis because the underlying theory is not sufficiently strong, giving rise to concerns about an oversized truth table. Let us assume that the truth table is indeed overspecified because median voter change is the only minimally sufficient condition. Since all configurations are covered by cases, it is possible to perform comparisons that are complemented by process tracing in order to determine whether a condition should enter into the analysis. A comparison of row A and B would let us infer that previous policy change is not causally relevant because the conjunct varies while the outcome is invariant. The two rows reduce to $MV \wedge MS$. We further conclude that *PC* is redundant in conjunction with $MV \wedge \lnot {MS}$ based on a comparison of C and D. A comparison of row B and D would lead to the conjunction $MV \wedge \lnot {PC}$ and a comparison of row A and C would yield the conjunction $MV \wedge PC$. Based on these insights, we would infer in a second round of comparisons centered on the two-way conjuncts that the presence and absence of *MS* and *PC* are redundant in conjunction with *MV*, letting us surmise that *MV* alone is sufficient. The comparative case studies would lead us to correctly determine *MS* and *PC* as causally unrelated to the outcome in combination with *MV*.

Given the functioning of the Quine–McCluskey algorithm that was described above, the way in which we arrive at this conclusion via case studies is *identical* with what the Quine-McCluskey algorithm would do if we were applying it to the data. The example highlights that charges against simulations because of their lack of case knowledge are not tenable because the singling out of causally irrelevant conjuncts via case studies already does the job of the truth table analysis. Imperfect case knowledge is a prerequisite for a follow-up QCA, for example because we are unable to perform all possible comparisons.

Building on this insight, two observations are in order at the end of this section. First, the same conclusion holds when a truth table involves unobserved conjunctions, i.e., limited empirical diversity. When an unobserved conjunction is needed to assess whether a conjunct is irrelevant, a comparative case study is replaced by a counterfactual inquiry on whether the unobserved conjunction would be associated with the outcome if a case was a member of the former. Counterfactual analyses before the truth table analysis replace the counterfactuals that are made in the course of the truth table analysis when the intermediate or parsimonious solution are generated (Ragin 2008, Chap. 8). Second, the arguments generalize to the application of the *Coincidence Analysis* algorithm (Baumgartner 2009) . This algorithm follows a different protocol for determining redundant conjuncts than the Quine–McCluskey algorithm, but the former would be equally pointless to apply if case studies allowed us to identify all causally irrelevant conjuncts before the truth table analysis.

## Sensitivity analyses are simulations

If we now follow my argument that case knowledge does not diminish modeling uncertainty in QCA of which the pre-selection of conditions is one instance, we are required to accommodate equally plausible modeling decisions in empirical QCA studies (Schneider and Wagemann 2012, Sect. 11.2).^7^ If we cannot defend a modeling decision over an alternative one, the natural solution is to run a sensitivity analysis (also called robustness test). For example, if we are uncertain about where exactly to set the consistency threshold for the assignment of outcome values when preparing the truth table, we should run multiple QCA with different consistency thresholds and determine whether the solution is sensitive to the selected parameter.^8^ The degree and sources of uncertainty vary across studies and requires different forms of sensitivity analyses (Skaaning 2011), but it is safe to argue all empirical research is confronted with some level of uncertainty.

Sensitivity analyses of the consequences of a modeling decision are the same as running a simulation. For a given empirical dataset, we manipulate a parameter of the design and compare how the QCA solution performs across all parameter specifications. An empirical researcher is simulating different datasets because each decision about a parameter such as the consistency threshold creates a new dataset that slightly differs from all others (although not necessarily leading to different solutions). What an empirical researcher is doing to assess the robustness of the results is indistinguishable from what we would do if we were approaching the same dataset from a methods perspective. In this instance, we would not care about the substantive conclusions supported by the QCA solutions. Instead, we would be interested in the robustness of the QCA results across different modeling decisions. In procedural terms, the important point is that a method-driven QCA researcher would pursue this goal by performing the same data and design manipulations as the empirical researcher. It follows that arguing against simulations for the assessment of QCA automatically refutes robustness tests in empirical research which is not a tenable position because modeling uncertainty is pervasive.

## Data-related reasons for simulations

### Limited diversity and counterfactuals

Limited diversity describes the phenomenon that possible conjunctions are not observed empirically. Counterfactuals are the instrument with which unobserved conjunctions are handled in empirical research. The implications of counterfactuals for simulations requires it to distinguish between the use of the Quine-McCluskey algorithm (henceforth QMC) on the one hand and Coincidence Analysis on the other (henceforth CNA). I first deal with QMC.

The way it was introduced in QCA, QMC allows it to derive three types of solutions depending on how one handles possible, but unobserved configurations that are usually called logical remainders (Ragin 2008, Chaps. 7–9). The *conservative* solution applies the one-difference rule to all observed conjunctions consistently linked to the outcome and excludes all others. It is usually argued that the conservative solution does not involve counterfactuals on remainders (Schneider and Wagemann 2012, p. 162), but this is wrong and can be demonstrated by following the distinction between primary and secondary counterfactuals (Steglich-Petersen 2012, pp. 118–119).

For purposes of illustration, suppose we are interested in explaining government termination due to the early resignation of the prime minister (*Y*). The conjunct of interest is ‘minority government’ (*X*) that, together with other conjuncts that do not matter here, is linked to *Y* because minority governments tend to be unstable. In the truth table, we have cases for the sufficient relation $X \Rightarrow Y$, but not for the association between $\lnot {X}$ and *Y*. When we derive the conservative solution that takes *X* as a non-redundant condition, we have to commit the primary counterfactual that $\lnot {X}$ is associated with $\lnot {Y}$. If we were not making this counterfactual, we would have to argue that $\lnot {X}$ would be associated with *Y* which would render *X* redundant and mean the same as making a simplifying assumption which is in discord with the nature of the conservative solution. This counterfactual is a primary counterfactual because we only ask whether *Y* would be present if *X* was absent. The fallacious reasoning about counterfactual-free conservative solutions implicitly refers to secondary counterfactuals because they ask what $\lnot {X}$ and $\lnot {Y}$ represent in conceptual and substantive terms. If *X* is a minority government, is $\lnot {X}$ a majority government? Or an oversized government? And would $\lnot {Y}$ be the defeat of the prime minister at a party convention, which would be another sign of instability? Or simply that the prime minister stays in power? These are questions secondary counterfactuals answer and which, in fact, do not have to be addressed in producing the conservative solution. However, a primary counterfactual is always made and explains why the conservative solution builds on counterfactual reasoning.

The *parsimonious* solution draws on observed configurations associated with *Y* and invokes counterfactuals on unobserved configurations. The counterfactuals on what the outcome of a case would be if a case was a member of a conjunction are made such that we obtain the most parsimonious superset of the conservative solution (Schneider and Wagemann 2012, Sect. 6.4). The *intermediate* solution sets theoretical constraints on the counterfactuals imposed on unobserved configurations. The constraints take the form of directional expectations that specify whether we expect the presence or absence of a conjunct to be associated with the outcome. The generation of all three solutions in one analysis is referred to as the ’standard analysis’.^9^

Corresponding with the case orientation of QCA, counterfactuals on unobserved configurations can be anchored in possible-world reasoning (Lewis 1973). Following the idea of possible worlds, counterfactuals on single cases requires us to consider what the outcome would be in a world that forms the closest-possible world to the actual one in which we live. Reference to possible worlds implies that the world could have developed differently, with the consequence that the world we live in is only one possible manifestation out of many (Menzel 2015).

Possible-world counterfactuals motivates simulations in two ways. First, on a philosophical dimension simulations allow us to evaluate how QCA performs in all possible worlds that could have come into existence. Second and being more relevant for empirical studies, we naturally do not know whether the counterfactuals we make are right or wrong because we make a causal argument for an unobserved empirical relationship in a possible world. Philosophical and social science work on counterfactuals developed an elaborated toolbox for the generation of counterfactuals (Lebow 2010; Lewis 1973; Stalnaker 2011), but establishing the truth value of counterfactual claims always remains the weak spot of possible-world reasoning.^10^ The uncertainty that underlies our counterfactuals can be addressed in simulations that assess the dependence of the QCA solution on the type of counterfactuals we make. The rejection of simulations for this purpose implies the claim that we can make counterfactuals that are very likely to be correct. This might be the case for selected empirical studies and counterfactuals, but this is a position which is hard to defend in general.^11^

In contrast to QMC, CNA does not invoke counterfactuals (Baumgartner 2009). CNA exclusively relies on the observed configurations and produces one solution which is equivalent to the parsimonious solution derived by QMC. If one prefers CNA over QMC and dispenses with counterfactuals, it is possible to deny a meaningful role for simulations in this respect. While a preference for CNA and QMC has implications for defensible positions one can take toward simulations, the remaining two data-related issues have similar implications for both algorithms.

### Sampling

Sampling is one of two research design features that motivate simulations for the evaluation of an empirical QCA study. The rationale for simulations is straightforward because the same modeling decision might play out differently in different samples. Since causal inference targets the population and not the sample, generating conclusions based on one sample would be a precarious strategy. The basis for causal inference becomes stronger if we assess the sensitivity of the QCA solution to our modeling decision across different samples. If we are unable to draw multiple samples from the population, which is the rule in empirical research, the simulation of multiple samples is a feasible alternative.

An objection might be that QCA was not designed for sampling-based analyses, which would be correct because the importance of carefully delineating a population of cases is a central issue in the literature (Ragin 2000, Chap. 2, 7). However, a look at empirical studies shows that this advice has not always been followed (Vis 2009). Depending on the research question, it might also not be possible to have a finite population because new cases are still being generated. For example, Schimmelfennig et al. (2006) are interested in the conditions of EU constitutionalization via formal constitutional decisions made at intergovernmental conferences. Schimmelfennig et al. carefully specify the cases of interest and delineate the population comprising 66 cases as of 2004 (Schimmelfennig et al. 2006, pp. 1169–1170). There is no theoretical rationale for taking the intergovernmental conference in 2004 as a temporal bound on the population, as this decision is driven by the need to stop collecting data in order to perform an empirical analysis.^12^ As a consequence, the 66 cases in the Schimmelfennig et al. (2006) study are a sample of a still increasing population. For QCA studies such as this that rely on samples, simulations are a viable tool for strengthening causal inference.

### Missing data

The third data-related reason for simulations in a given empirical study is missing data. The reasoning is similar to sampling. A QCA study with missings in the dataset relies on data that is different from the full dataset that we want to analyze from a population-based perspective. Regardless of the type of missingness (Allison 2001), one should take into account that a QCA of the observed data might lead us to come to different conclusions than a QCA on the full dataset. In the absence of any other reliable imputation strategy for QCA, for now at least, simulations constructing numerous complete datasets should be useful for assessing how sensitive the QCA solution is across them.

## Monte Carlo simulations give general insights into QCA

Empirical researchers are interested in the validity of the results derived from one dataset, while method-driven researchers are interested in the *general* performance of QCA. This distinction might be obvious, but it is crucial and conveys one important insight. A single empirical dataset that we make subject to sensitivity tests might demonstrate that the QCA solution is fully robust to alternative modeling decisions. This would be good news for the empirical study, but we cannot generalize from this that QCA works well in general. For empirical data, we do not know the data-generating process and correct modeling decisions, meaning that we cannot tell whether the robust solution is identical with the true solution. Furthermore, the empirical data might have turned out favorably by chance, i.e., the altering of model parameters for the given analysis is inconsequential for the solution. For another dataset, the solution might be highly sensitive to alternative modeling decisions. One-shot simulations of hypothetical data do not suffer from these problems because we model the data-generating process, but they are equally vulnerable to generalizing about QCA in general based on one dataset. Since we do not know whether the simulation produced a dataset that is favorable or unfavorable for QCA by chance, we should not infer anything from one-shot simulations.

Monte Carlo (MC) simulations are superior to simulations involving a single empirical study and one-shot simulations because a large number of simulations of, say, 1000 datasets will include single datasets that are favorable and unfavorable for QCA. For this reason, MC simulations give us a general and balanced picture about the sensitivity of QCA to a modeling decision or data-related feature instead of being dependent on a single dataset. The rejection of MC simulations means the rejection of general inquiries into the performance of a method, making it an open question as to how general assessments are to be made if not via MC simulations.

## Conclusion: the way forward involves Monte Carlo simulations

In recent years, simulations have become a widely used tool for the evaluation of QCA and triggered a discussion about their sense and non-sense. This article builds on the meta-debate and comes to the conclusion that the arguments against simulations are not tenable. Some of my arguments are specific and only apply to the Quine–McCluskey algorithm or empirical studies with specific data characteristics. However, I showed that the supposedly strongest charge against simulations, their absence of case knowledge, is self-defeating because it undermines the need for running a truth table analysis and the reason why one is doing a QCA. This is a general insight generalizing to QCA studies regardless of their design features and implemented algorithm.

I conclude that the debate about the performance of QCA should move from the meta-level to the discussion of *how* to model specific aspects of QCA in MC simulations. As I emphasized in the introduction, the claim that simulations are useful in principle does not mean that the existing simulations have been implemented in line with the principles of QCA. For example, Krogslund, Choi and Poertner run, among other things, simulations on the sensitivity of QCA solutions of three empirical studies to the chosen minimum and maximum consistency threshold for assigning values to the outcome of truth table rows (Krogslund et al. 2015). Per empirical study and selected frequency threshold, they sample 3000 pairs of inclusion scores. At first glance, this might seem impressive, but a second look reveals that the simulation is strongly overpowered. The maximum inclusion threshold is irrelevant regardless of the solution one derives with QMC because it does not influence which rows receive a “1” on the outcome or are treated as remainders. This means that the intersection of the minimum and maximum threshold is pointless. Of the remaining truth table analyses covering the entire range of scores for the minimum threshold, all that fall below the conventional minimum threshold of 0.75 are irrelevant. Taken together, only a small share of all 3000 truth table analyses offers relevant insights. If we focus only on the relevant simulations in the figures generated by Krogslund et al. we see that all results are more robust than reported by Krogslund et al.

Based on the premise that simulations are a valuable tool for assessing the performance of QCA in general and in specific empirical studies, there is sufficient work ahead in scrutinizing existing simulations and devising additional ones on hitherto unexplored issues such as the relative performance of different algorithms in the presence of modeling uncertainty.

## Footnotes

1 I distinguish between QCA as a set-relational technique and the algorithm that is used in the truth table analysis as one element that defines QCA.

2 For example, one can also rely on case-based insights for the validation of the chosen measures (Adcock and Collier 2001), but this is independent of the truth table analysis because the latter cannot yield insights on measurement validity.

3 Another problem involves cases that share the same configuration but display different outcomes, called contradictions. Exploratory case studies might resolve contradictions (Ragin 1987, pp. 113–118), but this needs to be done before the algorithm can be applied.

4 The complementary problem is underspecification, i.e., the exclusion of causally relevant conditions from the truth table.

5 ‘Conjunct’ denotes a condition that belongs to a conjunction of conditions.

6 The same arguments hold for multivalue sets, the use of which would increase the size of the truth table. Whether a configuration is fully consistently linked to the outcome or not is neither relevant nor is the number of cases per conjunction.

7 Other elements of QCA potentially introducing modeling uncertainty concern the shape of the population; concept formation and measurement; the choice of the set type, calibration thresholds and calibration function; the consistency threshold for the assignment of outcome values to truth table rows.

8 In my view, there is no unequivocal understanding of what a “robust” finding is. Existing simulations concerned with QCA solutions exclusively focus on whether one gets the same solution across different analyses. This is one possible criterion, but also the highest possible standard. Ultimately, what a robust result is depends on the research goal and might also pertain to the question of whether a single conjunction is always part of the solution.

9 My arguments extend to the enhanced standard analysis (ESA) and the theoretically enhanced standard analysis (TESA) proposed by Schneider and Wagemann (2012, Chap. 8).

10 In the QCA literature, the distinction between difficult and easy counterfactuals (Ragin 2008, Chaps. 7–9) and TESA (Schneider and Wagemann 2012, Chap. 8) address counterfactual reasoning, but do not offer criteria on how to establish good counterfactuals.

11 Possible-world semantics for single cases is not the only way to construct counterfactuals. The current major theory of causation is Woodward’s interventionist theory which relies on counterfactuals on the type level, i.e., a large-n setting in social science terms (Woodward 2003). The interventionist theory has received considerable attention in philosophy and the social sciences (Morgan and Winship 2014), but its type-level orientation does not fit with the case-based nature of QCA. The single-case orientation of possible-world reasoning and the case orientation of QCA achieve a much better fit and are therefore consider here.

12 See Rohlfing (2012, h. 9) for a discussion of types of bounds on populations.
